# Small vessel disease contributions to acute delirium: a pilot feasibility MRI study

**DOI:** 10.1093/ageing/afaf099

**Published:** 2025-04-16

**Authors:** Una Clancy, Yajun Cheng, Charlotte Jardine, Fergus Doubal, Alasdair M J MacLullich, Joanna M Wardlaw

**Affiliations:** Centre for Clinical Brain Sciences, University of Edinburgh, Edinburgh, UK; UK Dementia Research Institute, University of Edinburgh, Edinburgh, UK; Row Fogo Centre for Research into Ageing and the Brain, University of Edinburgh, Edinburgh, UK; Department of Neurology, West China Hospital, Sichuan University, Chengdu, China; Edinburgh Imaging, University of Edinburgh, Edinburgh, UK; Centre for Clinical Brain Sciences, University of Edinburgh, Edinburgh, UK; UK Dementia Research Institute, University of Edinburgh, Edinburgh, UK; Row Fogo Centre for Research into Ageing and the Brain, University of Edinburgh, Edinburgh, UK; Geriatric Medicine, University of Edinburgh MED, F1424, Royal Infirmary of Edinburgh 51, Little France Crescent, Edinburgh EH16 4SA, UK; Centre for Clinical Brain Sciences, University of Edinburgh, Edinburgh, UK; UK Dementia Research Institute, University of Edinburgh, Edinburgh, UK; Row Fogo Centre for Research into Ageing and the Brain, University of Edinburgh, Edinburgh, UK

**Keywords:** delirium, cerebral small vessel diseases, cerebrovascular disorders, magnetic resonance imaging, cognitive dysfunction, older people

## Abstract

**Background and aims:**

Delirium carries an eight-fold risk of future dementia. Small vessel disease (SVD), best seen on magnetic resonance imaging (MRI), increases delirium risk, yet delirium is understudied in MRI research. We aimed to determine MRI feasibility, tolerability, image usability and prevalence of SVD lesions in delirium.

**Methods:**

This case–control feasibility study performed MRI (3D T1/T2-weighted), fluid-attenuated inversion recovery, susceptibility-weighted and diffusion-weighted imaging (DWI) on 20 medical inpatients >65 years: 10 with delirium ≥3 weeks and 10 without delirium, matched for vascular risk, Clinical Frailty Scale (CFS) and cognition. We excluded acute stroke, agitation necessitating sedation, mobility assistance of >2 and MRI contraindications. We measured scan duration, tolerability, image usability, acute infarcts and SVD features. Six months later, we recorded CFS and cognitive diagnoses.

**Results:**

Mean age was 83.5 years (delirium 78.7 vs non-delirium 88.4); 13/20 were female; 17/20 had premorbid cognitive decline/impairment or dementia. Acquisition took mean 26.8 min. MRI was well tolerated in 16/20 (7/10 in delirium arm; 9/10 in non-delirium arm). Also, 4/20 had early scan termination, but 20/20 had clinically interpretable images. We detected DWI-hyperintense lesions in 3/10 (30%) with delirium (2/10 small subcortical and 1/10 cortical) and in 3/10 (30%) without delirium (2/10 small subcortical; 1/10 cortical). Mean white matter hyperintensity Fazekas score was 6 in delirium versus 4.5 without.

**Conclusions:**

MRI is feasible, usable and tolerable in delirium, and we detected DWI-hyperintense lesions in one-third of all study participants, regardless of delirium status. This study indicates acute vascular contributions, including SVD, to both delirium- and non-delirium–related presentations, supporting the need for larger studies.

## Key Points

- Delirium is understudied in magnetic resonance imaging (MRI) research.
- MRI is feasible, tolerable and interpretable during acute delirium.
- Diffusion-weighted imaging hyperintensities are present in 30% of frail inpatients with or without delirium.
- This pilot study provides insights into the potential role of acute vascular contributions to delirium.

## Introduction

Delirium is an acute disturbance in attention, awareness and cognition that affects a quarter of older inpatients [1, 2]. Delirium is associated with poor cognitive and functional outcomes [3] and carries an 8.7-fold risk of incident dementia in older people [4]. Persistent delirium, lasting weeks or months, occurs in 20%–40% of delirium patients and carries worse outcomes [5, 6]. Persistent delirium has been proposed as a marker for chronic cognitive disorders such as dementia [7].

Delirium aetiology is poorly understood and there is no specific treatment apart from addressing potential precipitants. Brain vascular dysfunction may contribute to delirium pathogenesis [1]. Delirium risk is higher in individuals with more severe cerebral small vessel disease (SVD) [8], a dysfunction of the perforating arterioles, capillaries and venules that is a common cause of dementia and is prevalent in older people [9]. In stroke patients, infection increases dementia risk following an episode of delirium in patients with SVD [10]. It is not clear, however, whether delirium is a mediating factor between SVD and dementia, or whether there exists a temporal sequence of SVD followed by delirium followed by dementia*.* Individuals with background SVD may have ‘frailer’ brains, where areas of vascular dysfunction may be more susceptible to acute impairment following systemic inflammation [11].

Patients with delirium have been understudied in magnetic resonance imaging (MRI)–based research. In clinical practice, computed tomography (CT) is the primary neuroimaging modality in patients with delirium, yielding acute intracranial findings in up to 14.5% of patients [12]. On CT, acute intracranial pathology has been predicted not only by focal neurological deficits, but also by recent falls or decreased consciousness, both common geriatric medicine presentations [12]. However, MRI is far more sensitive than CT for detecting acute infarcts [13], especially smaller subcortical lesions, and many lesions retain MRI features to indicate that they are recent for several weeks after onset. However, few MRI studies have imaged patients during an acute delirium episode, and most have focused on functional [14–17] rather than structural MRI, which is sensitive to acute and chronic vascular lesions. Two studies have evaluated structural MRI during a delirium episode, one in an intensive care population with septic shock [18] and another in a retrospective clinical population [19]. Several delirium studies have assessed chronic rather than acute SVD features on MRI [8, 20] in mostly surgical populations where imaging took place prior to surgery. We are not aware of any prospective structural MRI studies of older medical inpatients performed during a delirium episode. We hypothesise that by detecting vascular abnormalities on structural neuroimaging, MRI carries potential for greater understanding of delirium aetiology outwith standard investigations.

This pilot feasibility study aims to determine the following in acute medical inpatients with and without persistent delirium: (1) the feasibility and tolerability of performing brain MRI; (2) the usability of MRI data; (3) the prevalence of acute and chronic SVD lesions on MRI; and (4) the feasibility of establishing cognitive and functional status by 6-month telephone follow-up.

## Methods

The Brain Imaging in Delirium study is a prospective, observational, case–control pilot feasibility study of older medical inpatients admitted to hospital with and without persistent delirium. We collected clinical and imaging data at baseline (≥3 weeks into delirium episode and/or hospital admission) and cognitive and functional status 6 months later by telephone.

We recruited and performed structural brain MRI on 10 medical inpatients ≥65 years with persistent delirium (50%) and 10 control inpatients without delirium (50%) who were admitted to the Royal Infirmary of Edinburgh from December 2022 to December 2023. We recruited participants from geriatric and general medical wards. Delirium and non-delirium participants were matched for Clinical Frailty Scale (CFS) [21], cognitive status and vascular risk factors. Persistent delirium was defined as an admission 4AT score ≥4 *and* 3-week 4AT score ≥4. We included patients in the delirium group if they had had CT during the current admission to exclude intracranial haemorrhage, infection, mass or infarct. The 10 participants without delirium were defined as having no clinical history suggestive of delirium documented during the current admission and no current evidence of delirium according to the Diagnostic and Statistical Manual of Mental Disorders, Fifth Edition (DSM-5) criteria. We scanned participants ≥3 weeks from admission to hospital. Although there is no agreed definition of persistent delirium, our rationale for the ≥3-week definition of persistent delirium is that 3 weeks is longer (mean + 2SD) than the mean delirium duration of 7 days [22], this allows sufficient time for treatment of any identifiable delirium precipitants, and imaging after this time window is less sensitive for detecting imaging features that may indicate a recent infarct.

We excluded patients if they had evidence of intracranial haemorrhage, infection, mass or obvious acute infarct on CT; were anticipated to require assistance of >2 staff to transfer from wheelchair to bed/scanner; were regarded by their clinical team as too agitated to undergo brain MRI or had required sedation in the preceding 48 h; had MRI contraindications; had ongoing medical interventions that would preclude MRI, e.g. oxygen supplementation; or had other major neurological conditions affecting brain imaging findings, e.g. tumour. Premorbid cognitive impairment was not an exclusion criterion.

Capacity to give informed consent was not a requirement for either arm of the study. In cases where participants lacked capacity, we asked their Nearest Relative/Guardian or Welfare Attorney to provide informed consent on their behalf under the Adults with Incapacity (Scotland) Act 2000.

### Study assessments

#### Clinical data

We collected most clinical variables from the electronic patient record (EPR). We recorded demographic details, pre-admission and current CFS, reason for hospitalisation, documented delirium causes, date of first 4AT score ≥4, date of last 4AT score ≥4 (or in the case of controls, documentation and clinician-confirmed absence of delirium), duration of delirium and vascular risk factors including previous stroke/transient ischaemic attack (TIA). We used the short-form IQCODE [23], using modified wording [3] to capture pre-hospital cognition versus 10 years ago. We recorded cognitive status as (1) dementia, (2) mild cognitive impairment (MCI), (3) documented history or concern of pre-admission cognitive decline in clinical notes that had not yet been investigated by a memory service, or (4) no cognitive concerns. Finally, we used the abbreviated version of the Delirium Motor Subtyping Scale [24].

Six months later, we telephoned living participants and/or their Nearest Relative/Guardian or Welfare Attorney to repeat CFS and record any incident cognitive impairment diagnoses, i.e. mild cognitive impairment or dementia. We collected mortality data from EPR.

#### Brain MRI

Participants attended a 25-min structural MRI ≥3 weeks into their admission using a protocol designed for acute stroke imaging on a 3 T (Siemens Prisma) MRI. All participants underwent a 5-min fast scan protocol consisting of diffusion-weighted imaging (DWI), which is sensitive to detecting acute infarcts, gradient recall echo and 2D fluid-attenuated inversion recovery (FLAIR). If this was tolerated, participants then underwent a 20-min standard protocol consisting of core structural sequences prioritised as follows to maximise detection of acute lesions in the event of early scan termination: DWI, 3D FLAIR, 3D T1-weighted, T2-weighted and susceptibility-weighted imaging. Our rationale for choosing a fast followed by standard protocol was to facilitate early identification of participant comfort levels, to maximise tolerability, and to determine feasibility and clinical utility of both approaches. Details of the standard stroke imaging protocol are outlined elsewhere [25].

The study team transported participants from the ward to the scanner, ensured that participants were comfortable prior the scan checking verbal and non-verbal cues, provided participants with an emergency button to stop the scan if needed, provided earplugs and padding for comfort, and spoke to participants at regular intervals to check for distress.

We recorded (1) duration of scan acquisition tolerated (minutes) and duration spent in scanner (minutes); (2) number of, and reason for, repeated sequences; (3) patient-rated and informant-rated tolerability as used in previous studies; and (4) clinician- and radiographer-rated tolerability.

We assessed the proportion of usable imaging data and rated imaging features using a dedicated pro forma (see Appendix 1 in the Supplementary Data section for MRI grading form). This pro forma was an adaptation of rating tools developed at our centre for previous studies in stroke [25, 26] and community-dwelling older populations [27], which laid the foundation for international image rating criteria [28, 29]. We visually rated acute and chronic infarcts and individual SVD features using STandards for ReportIng Vascular changes on nEuroimaging criteria [28, 29]. We defined an acute lesion as a recent infarct visible on DWI or FLAIR and/or T2-weighted sequences. We assessed the following SVD features: atrophy [30], Fazekas white matter hyperintensity score [31], perivascular space count [32], number of microbleeds [33], number of lacunes and summary SVD score [29, 34]. We also checked for the presence of non-vascular imaging abnormalities. All image analysis was supervised by a neuroradiologist (J.M.W.) who was blinded to details of clinical presentation.

### Statistical analysis

We stratified participants by delirium versus non-delirium status. The sample size of the present pilot analysis is intended to provide preliminary data and is not designed to test associations adjusted for key variables. We report the mean (SD) or median [interquartile range (IQR)], and *n* (%) for all data. We describe the proportion of patients who achieved full versus partial scan; patient and radiographer/clinician-rated tolerability; proportion with usable imaging data; and proportion with acute and chronic infarcts according to infarct subtype, summary SVD score, and 6-month cognitive and functional status. We used the Shapiro–Wilk test to test for normality: age and IQCODE were normally distributed whilst SVD features were not. For normally distributed variables, we used the unpaired two-samples *T*-test (*t*). For non-normal distributions, we used Kruskal–Wallis (KW) test. For comparison of group proportions, we used Fisher’s exact test.

We analysed data using R (http://www.R-project.org/).

Ethical approval was granted by South East Scotland Research Ethics Committee (22/SS/0076).

## Results

We recruited 20 participants of mean age 83.5 (SD 7.8) years; delirium 78.7 years versus non-delirium 88.4 years. Of 20 participants, 17 (85%) had premorbid cognitive decline/impairment or dementia (Table 2). Mean delirium duration was 28 (SD 13) days in the delirium arm. Delirium was subtyped as hypoactive in 4/10, hyperactive in 2/10 and mixed in 4/10. We describe population characteristics in Table 1. Admission diagnoses are outlined in Appendix 2 of the Supplementary Data section, showing that non-delirium participants were predominantly admitted with falls and/or declining mobility. We achieved 6-month follow-up in 19/20 participants and for the remaining participant we acquired EPR data on mortality and cognitive status.

**Table 1:** Population characteristics

	Persistent delirium arm (*n* = 10)	Non-delirium arm (*n* = 10)	Total population (*n* = 20)	
Age, years (mean, SD) Shapiro–Wilk normality test *P* = .61	78.72 (7.02)	88.43 (5.30)	83.57 (7.89)	*t* = −3.3, *P* = .004
Female sex, *n* (%)	5 (50)	8 (80)	13 (65)	*P* = .34
Pre-admission CFS, mean (SD)	5 (1.3)	6 (0.6)	5 (1.1)	*t* = −1.1, *P* = .25
CFS on MRI scan date, mean (SD)	6 (0.9)	6 (0.5)	6 (0.8)	*t* = 1.4, *P* = .17
Delirium duration (mean, days)	28 (13)	–	–	
BP on MRI date, systolic (mean, mmHg)	130	126	128	*t* = 0.3, *P* = .71
BP on MRI date, diastolic (mean, mmHg)	75	72	73	*t* = 0.7, *P* = .46
Head CT during admission, *n* (%)	10 (100)	5 (50)	15 (75)	*P* = .03
**Vascular risk factors**				
TIA, *n* (%)	2 (20)	4 (40)	6 (30)	*P* = .62
Stroke, *n* (%)	2 (20)	2 (20)	4 (20)	*P* > .99
Ischaemic heart disease, *n* (%)	4 (40)	1 (10)	5 (25)	*P* = .30
Diabetes, *n* (%)	3 (30)	2 (20)	5 (25)	*P* > .99
Hypertension, *n* (%)	6 (60)	7 (70)	13 (65)	*P* > .99
Atrial fibrillation, *n* (%)	4 (40)	3 (30)	7 (35)	*P* > .99
Hypercholesterolaemia, *n* (%)	2 (20)	0 (0)	2 (10)	*P* = .47
Cardiac failure, *n* (%)	1 (10)	2 (20)	3 (15)	*P* > .99
Valvular abnormalities, *n* (%)	2 (20)	1 (10)	3 (15)	*P* > .99
Ever smoker, *n* (%)	7 (70)	3 (30)	10 (50)	*P* = .17

SD, standard deviation; CFS, clinical frailty scale; BP, blood pressure; MRI, magnetic resonance imaging; CT, computed tomography; TIA, transient ischaemic attack.

**Table 2:** Population cognitive status

	Persistent delirium arm (*n* = 10)	Non-delirium arm (*n* = 10)	Total population (*n* = 20)	
IQCODE mean (missing *n* = 4; 2 each arm) Shapiro–Wilk normality test *P* = .82	4.20	3.98	4.09	*t* = 0.92, *P* = .37
Dementia diagnosis, *n* (%)	1 (VaD) (10)	2 (VaD) (20)	3 (15)	*P* = .58
MCI diagnosis, *n* (%)	3 (30)	3 (30)	6 (30)	*P* > .99
Clinical concern of cognitive decline but no formal diagnosis, *n* (%)	4 (40)	4 (40)	8 (40)	*P* > .99
No cognitive concerns/diagnoses, *n* (%)	2 (20)	1 (10)	3 (15)	*P* > .99

Delirium symptom descriptors included on head CT requests for the delirium arm included ‘confusion/confused’ in 7/10, ‘drowsy’ in 2/10 and no delirium descriptor on CT head request for 1/10. Other descriptors in the same patients included ‘distressed’, ‘less responsive’, ‘difficulty finding words’ and ‘recent short term memory loss’. The hypoactive group was described as a mixture of ‘confused’ or ‘drowsy’, the hyperactive group was described as ‘confused’ only and the mixed group was mostly described as ‘confusion’.

MRI was well tolerated in 16/20 (7/10 in delirium arm vs 9/10 in non-delirium arm, *P* = .58). Mean scan acquisition took 26.8 (SD 7.7) min. Scans were terminated early in 5/20 (25%), with mean scan acquisition 16.4 (SD 7.6) min in the early termination group, but 20/20 participants had clinically interpretable images (Table 3).

**Table 3:** Scan factors and imaging abnormalities

	Persistent delirium arm (*n* = 10)	Non-delirium arm (*n* = 10)	Total population	
**Scan factors**				
Scan acquisition time, minutes, mean (SD)	25.8 (9.8)	27.8 (4.6)	26.8 (7.7)	*t* = −0.55, *P* = .58
Time in scanner, minutes, mean (SD)	30.6 (8.8)	33.6 (4.8)	32.1 (7.2)	*t* = −0.89, *P* = .38
Full structural scan protocol completed, *n* (%)	7 (70)	8 (80)	15 (75)	*P* > .99
Clinically interpretable images, *n* (%)	10 (100)	10 (100)	20 (100)	*P* > .99
Patient self-rating of scan as well tolerated or very well tolerated, *n* (%)	7 (70)	9 (90)	17 (85)	*P* = .58
Clinician/radiographer rating of scan as well tolerated or very well tolerated, *n* (%)	9 (90)	9 (90)	18	*P* > .99
**Imaging abnormalities**				
DWI-hyperintense lesion on MRI (yes)	3 (30)	3 (30)	6 (30)	*P* > .99
DWI-hyperintense *or* recent FLAIR lesion (yes)	4 (40)	3 (30)	7 (35)	*P* > .99
Fazekas periventricular WMH, median (IQR)	3 (3–3)	2.5 (2–3)	3 (2–3)	KW 1.6, *P* = .19
Fazekas deep WMH, median (IQR)	3 (2.25–3)	2 (2–3)	3 (2–3)	KW 1.0, *P* = .30
Fazekas total WMH, median (IQR)	6 (5.25–6)	4.5 (4–6)	6 (4–6)	KW 1.5, *P* = .21
Lacunes, median number (IQR)	0.5 (0–2.75)	2.5 (1.0–4.75)	1.5 (0–4.25)	KW 1.7, *P* = .19
Basal ganglia perivascular spaces rating, median (IQR)	2 (1.25–2.0)	3 (2–3)	2 (2–3)	KW 4.0, *P* = .04
Centrum semiovale perivascular spaces rating, median (IQR)	2 (2.0–2.75)	2 (2.0–2.75)	2 (2–3)	KW 0, *P* > .99
Cerebral microbleed number, median (IQR)	0 (0–0.75)	0 (0–1)	0 (0–1)	KW 0.03, *P* = .85
Deep atrophy rating, median (IQR)	5.5 (4.25–6)	5.5 (4.25–6)	5.5 (4–6))	KW 0.04, *P* = .83
Superficial atrophy rating, median (IQR)	4 (3.0–5.75)	5 (4–6)	5 (3–6)	KW 1.27, *P* = .25
Summary SVD score, median (IQR)	2.5 (1.0–3.75)	3 (3.0–3.75)	3 (2.77–4)	KW 1.9, *P* = .15
Old infarct number, median (IQR)	2 (0.25–3.75)	3.5 (2.0–5.75)	2 (1.75–5.25)	KW 1.0, *P* = .31
Old small subcortical infarct number, median (IQR)	0.5 (0–2.75)	2.5 (1.0–4.75)	1.5 (0–4.25)	KW 1.7, *P* = .19
Old cortical infarct number, median (IQR)	0 (0–0.75)	1 (0–1)	0 (0–1)	KW 0.77, *P* = .37

**Figure 1:**
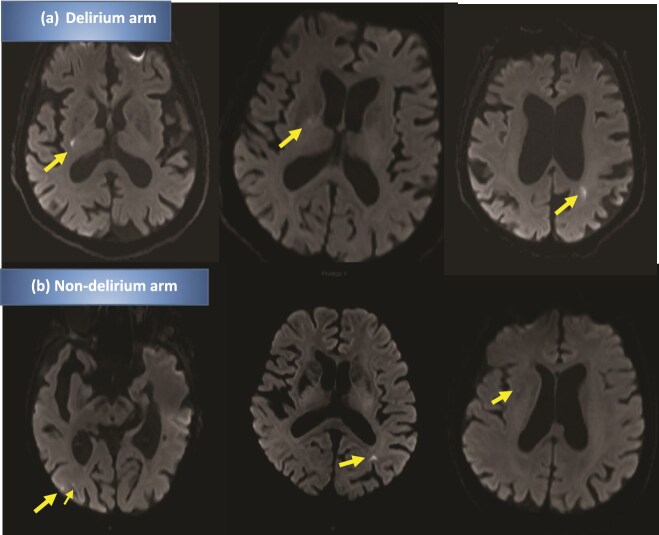
DWI-hyperintense lesions on MRI in (a) delirium arm and (b) non-delirium arm.

**Figure 2:**
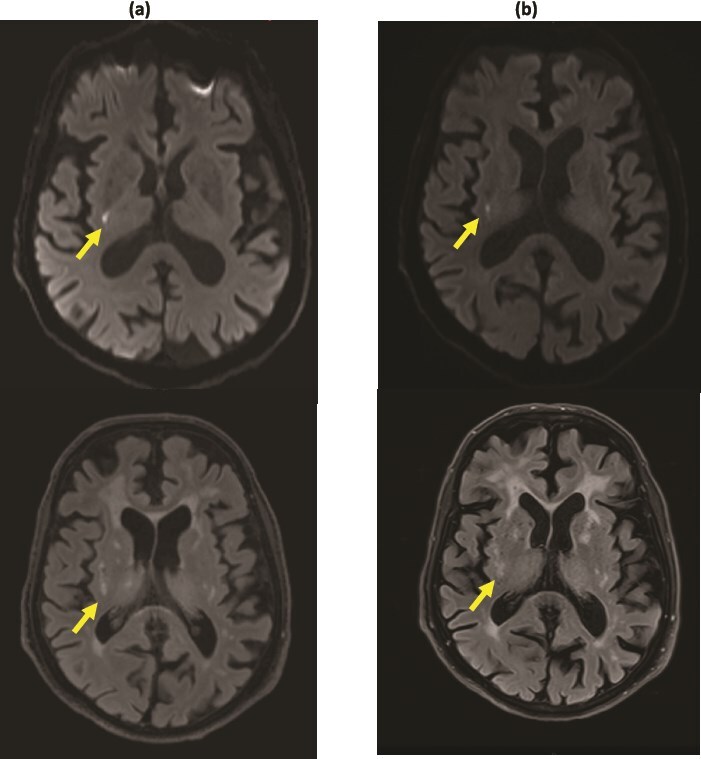
Translatable to clinical practice? DWI-hyperintense lesion shown in the same participant on (a) the 20-min standard MRI protocol with corresponding lesion on T2 FLAIR sequence vs (b) the 5-min fast MRI protocol with corresponding lesion on T2 FLAIR sequence.

We detected acute DWI-hyperintense lesions in 3/10 (30%) of the delirium arm and in 3/10 (30%) in the non-delirium arm (Figure 1), *P* > .99. Acute lesions in the delirium arm were located in the internal capsule, lentiform nucleus and centrum semiovale/posterior borderzone (two lesions in one participant). Acute lesions in the non-delirium arm were located in the juxtacortical parietal white matter, external capsule/cerebellar cortex (two lesions in one participant) and occipital/parietal cortex (two lesions in one participant). In all six participants with acute lesions, DWI-hyperintense lesions were detectable on both the fast 5-min MRI protocol and the standard 20-min MRI protocol (Figure 2). One further participant in the delirium arm had features consistent with subacute infarct on FLAIR (DWI-negative).

The median summary SVD score was 2.5 (IQR 1.0–3.7) in participants with delirium versus 3 (IQR 3.0–3.7) without delirium, KW 1.0, *P* = .15. The median total WMH Fazekas score was 6 (5.2–6.0) in participants with versus 4.5 (4–6) without delirium, KW 1.5, *P* = .21. The median number of chronic infarcts was 2 (0–2.75) in the delirium arm versus 3.5 (2.0–5.75) in the non-delirium arm, KW 1.0, *P* = .31. We outline chronic SVD features, summary SVD score and old infarct subtypes in Table 3.

At 6-month follow-up, 5/20 (25%) participants had died and of the remainder, 9/15 (60%) had received an incident diagnosis of mild cognitive impairment or dementia and 7/14 (50%) had worsening CFS. We outline 6-month cognitive and functional outcomes according to delirium status in Appendix 3 and according to acute DWI hyperintensities in Appendix 4 of the Supplementary Data section.

## Discussion

In this mostly cognitively impaired population with moderate–severe background SVD and without acute stroke syndromes, we found that MRI is feasible, tolerable and interpretable during the acute delirium episode. We detected DWI-hyperintense lesions in 30% of patients overall, both with and without delirium, which are equally detectable on a fast 5-min MRI versus a 25-min standard MRI protocol (Figure 2), showing strong potential for clinical translation to acute delirium populations. In this small sample, we found that WMH burden was higher but summary SVD score was lower in the delirium group. We also establish the feasibility of determining cognitive and functional status by 6-month telephone follow-up after delirium. This pilot study provides insights into the potential role of acute vascular contributions, including SVD, to delirium and mobility-related presentations.

Previous work showed that small subcortical lesions located in the internal capsule are most likely to cause typical stroke symptoms since motor and sensory function are densely represented in the corticospinal tracts, whilst lesions located in other subcortical regions are more likely to result in so-called ‘silent’ stroke or atypical stroke symptoms [35]. Most acute lesions in our study’s population were located outside the corticospinal tracts, which may explain atypical clinical presentations with delirium rather than stroke. Moreover, our cognitively impaired population may lack the ability to perceive, recall or communicate stroke symptoms, and our clinical experience is that focal neurological deficits are more difficult to elicit in cognitively impaired populations, meaning that traditional stroke syndromes may be easily missed.

The 30% prevalence of DWI-hyperintense lesions in the non-delirium group may reflect that this group’s primary complaint was mobility related, i.e. acute lesions may have precipitated syndromes of acute balance impairment without classic ataxia or unilateral motor symptoms that might ordinarily trigger a stroke review. Alternatively, acute infarcts in this population may reflect the high proportion of participants with cognitive impairment in both arms. It is unclear whether the acute brain changes reflect acute cognitive and physical deteriorations resulting in hospital admissions versus a more dynamic vascular element to chronic cognitive impairment than was previously thought. A serial imaging study would help to address this question. The summary SVD score was higher in the delirium arm. However, the WMH score was higher in the delirium arm. This suggests that WMH, rather than other SVD features, may be the primary SVD feature linked to delirium.

This study builds on work showing that chronic SVD features increase future delirium risk almost three-fold [8]. The high prevalence of DWI-hyperintense lesions in this population with multifactorial delirium elements including infection (Appendix 2 of the Supplementary Data section) adds to recent evidence showing that infection increases dementia risk following delirium in SVD [10]. Our findings highlights a novel acute cerebrovascular contribution to unscheduled care presentations in older people. SVD is increasingly recognised as a contributor to multi-domain impairments including gait, mood, cognitive decline, institutionalisation [36], mortality [37] and, now, acute healthcare presentations including delirium. Current delirium guidelines recommend that neuroimaging should be considered in patients with non-resolving delirium without clear aetiology [38]. Although our study’s findings need to be replicated in larger populations before we can recommend changes to clinical management, our findings add strength to guideline recommendations, highlight the value of MRI versus CT in detecting small acute vascular lesions and support MRI for persistent delirium even after a clear cause has been identified and treated.

The association between SVD and chronic cognitive impairment is well established [39]. However, it is not yet clear whether acute SVD lesions may precipitate episodes of delirium, initiating a cascade of deteriorating cognition long term, or alternatively, may be a manifestation of a prolonged inflammatory state. Systemic inflammation including infection is a key precipitant of both delirium and mobility impairment. Impaired blood–brain barrier permeability in patients with SVD [40] may increase susceptibility to circulating inflammatory mediators [41]. Regardless of whether SVD may be a precipitant or manifestation of delirium, the potential role of acute vascular lesions in delirium and declining mobility needs to be quantified in larger studies as it might explain why delirium and gait disorders are strongly linked to both the presence and development of dementia. Future research should perform serial longitudinal MRI to better determine whether acute infarcts may be a cause versus consequence of delirium.

The study strengths include a generalisable population of older medical inpatients with a mean pre-admission CFS of 5, a cohort that have been neglected in delirium MRI research. We acquired interpretable images in 20/20 (100%) participants. We prioritised specific sequences to maximise the detection of acute lesions in the event of attrition.

The study had limitations. This small study was not powered to assess differences between delirium and non-delirium arms, so our results should be interpreted cautiously. We excluded individuals who were agitated, which may have underrepresented individuals with hyperactive delirium. However, we chose this exclusion criterion to minimise participant risk, ensure comfort, reflect clinical practice and maximise image quality. Moreover, this is a novel MRI population aiming to strike a balance between patient comfort and successful image acquisition, the results will inform future research approaches in the wider population and mixed delirium was well represented. Although the 4AT has good diagnostic test accuracy for delirium, with specificity of 89%, specificity falls to 65%–79% in patients with delirium superimposed on dementia [42]. Consequently, given the high degree of premorbid cognitive impairment, it is possible that some patients in the delirium arm were misclassified: further research is needed in this group.

Patients with delirium were 10 years younger. We propose that this was due to survival bias in the persistent delirium group that survived to inclusion in this study (3 weeks) as delirium carries a 5.5-fold 30-day mortality risk [43]. Since age is a key risk factor for WMH, it is possible that the non-delirium group had a disproportionally high SVD burden due to their older age. Moreover, vascular dementia diagnoses were higher in this group so these preliminary findings should be interpreted with caution, as should any deductions about the influence of WMH extent/location. A study strength is a supporting foundation for much-needed larger studies.

This study proves the feasibility of performing MRI in an underrepresented group of older adults with delirium. This finding is translatable to clinical practice in delirium cases where MRI is clinically indicated, providing reassurance to clinicians that MRI is tolerable and that interpretable images are achievable. The findings give better insight into to the role of vascular lesions in delirium. The study findings are clinically translatable, showing that a fast 5-min MRI scan can detect acute DWI hyperintensities in patients with delirium. This has potential implications for delirium investigation in resource-constrained settings. Moreover, this study provides preliminary findings on the vascular contribution to persistent delirium, a condition that is poorly understood and is linked to incident dementia. Future larger studies should assess how strategic lesion location relates to specific delirium symptoms and subtypes, e.g. whether a higher burden of brainstem WMH corresponds more with a hypoactive picture. The potential role of acute vascular lesions in delirium needs to be quantified further as it may explain why delirium is strongly linked to both the presence and development of dementia, paving the way for future research.

## Supplementary Material

aa-24-1463-File002_afaf099
