# Supplementary material for: Small vessel disease contributions to acute delirium: a pilot feasibility MRI study
Source: Age Ageing. 2025 Apr 16;54(4):afaf099. doi: 10.1093/ageing/afaf099 (PMC12001778; doi:10.1093/ageing/afaf099)
Supplement: aa-24-1463-File002_afaf099 [file aa-24-1463-file002_afaf099.docx]

Appendix 1 MR grading form for Brain Imaging Delirium: Page 2-4

Appendix 2: Admission diagnoses: Page 5

Appendix 3: Six-month cognitive and functional outcomes according to delirium status: Page 6

Appendix 4: Six-month cognitive and functional outcomes according to presence or absence of acute infarct on brain MRI: Page 6

**Appendix 1 MR grading form for Brain Imaging Delirium**

Study ID…………….. ……. Reader……………

MR Sequences (circle) – T2 / FLAIR / GRE / T1/ DWI/SWAN or SWI/ CUBE/other

**Recent infarct**

(by age and location) present – **Y**es/ **N**o

**Sequence on which lesion present**: DWI : **Y**es/ **N**o FLAIR/T2 : **Y**es**/ N**o

Lesion Code*_(localization)_……… Side………. Shape: **t**ubular/**o**val-round…………

_If lacunar:_

Appearance now (code): _______

­­­­­­­

***Index lesion appearance codes***

| ***I. completely cavitated***  *visible on T2, FLAIR & T1* | ***II. partially cavitated***  *a) lacy*  *b) hole+large WMH rim*  *c) FLAIR cavity but not=CSF*  *d) FLAIR=WMH T2=cavity*  *e) visible on T2 not FLAIR*  *^$^If II, use supplementary codes e.g. IIc* | ***III. not cavitated (WML-like)***  ***IV. disappeared***    ***V. become visible*** |
| --- | --- | --- |

_If lacunar:_

Lesion diameter (mm): axial a:____ b: _ cran-caud:______ Shape: **t**ubular/**o**val-round____

**Old infarcts (including lacunar):** Y / N

1 2 3 4 5 6 7 8

**L**eft / **R**ight

Code*_(localization)_

**I**nfarct / **H**aemorrhage

If lacunar, cavitated Y/N

**Number of lacunes (not including any recent)**: ______

_(ovoid, >3mm , including those coded as ‘infarcts’ above but_ _excluding the index stroke)_

****Infarct codes – for acute/relevant or old***

| ***MCA cortical***  *1 small cortical*  *2 basal ganglia*  *3 subcortical*  *4 ant half periph MCA^#^*  *5 post half periph MCA^#^*  *6 whole peripheral MCA*  *7 whole periph + lat BG*  *8 whole MCA territory*  *^#^note may include some of lateral basal ganglia* | ***Other cortical***  *9 < half ACA*  *10 > half ACA*  *11 < half PCA*  *12 > half PCA*  *14 posterior BZ*  *13 anterior BZ* | ***Lacunar*****  *15 int & ext capsules/lent nucleus*  *a) internal capsule*  *b) external capsule*  *c) lentiform nucleus*  *16 internal border zone*  *17 centrum semiovale*  *18 thalamus*  *19 brainstem a) pons; b) medulla c) midbrain*  *25 cerebellum*  *26 optic radiation*  *27 splenium of corpus callosum*  *28 anterior frontal*  *29 anterior temporal*  *30 juxta-cortical*  *31 other* | ***Cerebellum***  *20 small cortical*  *21 <1/2 hemisphere*  *22 >1/2 hemisphere*  ***Brainstem***  *23 small (i.e. <1/2 medulla)*  *24 extensive (>1/2 medulla)* |
| --- | --- | --- | --- |

**Other abnormalities / additional comments**…………………………………………………………

………………………………………………………………………………………………………………

**White matter hyperintensities**

**Fazekas** *0 / 1 / 2 / 3* R L

Periventricular

Deep

**Perivascular spaces**  *0 = none / 1 = <10 / 2 = 11-20 / 3 = 21-40 / 4 = >40 _(^scores for PVS)_*

R L

Basal ganglia

Centrum semiovale

**Microbleeds:** R _(Indicate number)_ L _(Indicate number)_

Grey/white matter junction

Deep white matter

Basal ganglia

Internal/external capsule

Thalamus

Posterior fossa

**Atrophy** (circle response; use normative age template)

Deep atrophy: ______ **1**(<25^th^)/ **2**(25-50^th^)/ **3** (50-75^th^)/ **4** (75-95^th^)/ **5** (>95^th^) / >>5 use **6**

Superficial atrophy: ______ **1**(<25^th^)/ **2**(25-50^th^)/ **3** (50-75^th^)/ **4** (75-95^th^)/ **5** (>95^th^) / >>5 use **6**

**Comparison of fast vs standard sequence**

|  | **Fast** | **Standard** |
| --- | --- | --- |
| Acute infarct visible | Y / N / NA | Y / N / NA |
| Old infarcts visible | Y / N | Y / N |

______________________________________________________________________________________________

**This rating pro forma has been adapted from image rating tools used in the Mild Stroke Study 3,^1^ Mild Stroke Study 2,^2^ Mild Stroke Study 1,^3^ Lothian Birth Cohort 1936,^4^ and is linked to the STandards for ReportIng Vascular changes on nEuroimaging criteria^5,6^**

1. Clancy U, Garcia DJ, Stringer MS, et al.; Rationale and design of a longitudinal study of cerebral small vessel diseases, clinical and imaging outcomes in patients presenting with mild ischaemic stroke: Mild Stroke Study 3. *European Stroke Journal*;**2020**(0):2396987320929617. doi: 10.1177/2396987320929617.
2. Makin SD, Doubal FN, Dennis MS, et al.; Clinically Confirmed Stroke With Negative Diffusion-Weighted Imaging Magnetic Resonance Imaging: Longitudinal Study of Clinical Outcomes, Stroke Recurrence, and Systematic Review. *Stroke* 2015;**46**(11):3142-8. doi: 10.1161/strokeaha.115.010665.
3. Wardlaw JM, Doubal F, Armitage P, Chappell F, Carpenter T, Muñoz Maniega S, Farrall A, Sudlow C, Dennis M, Dhillon B. Lacunar stroke is associated with diffuse blood-brain barrier dysfunction. Ann Neurol. 2009 Feb;65(2):194-202. doi: 10.1002/ana.21549. PMID: 19260033.
4. Wardlaw JM, Bastin ME, Valdés Hernández MC, et al.; Brain aging, cognition in youth and old age and vascular disease in the Lothian Birth Cohort 1936: rationale, design and methodology of the imaging protocol. *Int J Stroke* 2011;**6**(6):547-59. doi: 10.1111/j.1747-4949.2011.00683.x.
5. Wardlaw JM, Smith EE, Biessels GJ, et al.; Neuroimaging standards for research into small vessel disease and its contribution to ageing and neurodegeneration. *Lancet Neurol* 2013;**12**(8):822-38. doi: 10.1016/s1474-4422(13)70124-8.
6. Duering M, Biessels GJ, Brodtmann A, et al.; Neuroimaging standards for research into small vessel disease-advances since 2013. *Lancet Neurol* 2023. doi: 10.1016/s1474-4422(23)00131-x.

| **Delirium group** |
| --- |
| Pneumonia; Atrial fibrillation with rapid ventricular response; Delirium |
| Delirium; Type 2 respiratory failure and pneumonia requiring Non-invasive ventilation on background of Chronic Obstructive Pulmonary Disease; ?underlying cognitive impairment vs dementia |
| Recurrent falls, delirium on background of possible cognitive impairment, infection (urine vs skin), opioid medication |
| Delirium on background of Parkinson's Disease and cognitive impairment; Urinary retention secondary to constipation; leg ulcer ?infected/?inflamed; New sertraline, constipation |
| Delirium on background of mild cognitive impairment with negative delirium screen; Acute Kidney Injury; social concerns about managing at home, falls |
| Fall with head injury; Urinary retention; UTI; Hypoactive delirium |
| Fall; Probable community acquired pneumonia vs Klebsiella UTI; Constipation, sensitivity to morphine sulphate; Delirium |
| Fall; Poorly controlled heart failure; Chronic leg ulcers with secondary infection; Delirium and chronic cognitive decline on background of mild cognitive impairment; new fast atrial fibrillation, infection unknown origin, E. coli UTI, bilateral pneumonia |
| Community Acquired Pneumonia; delirium on background of suspected longstanding cognitive impairment; Poor mobility; Fall and vomit |
| Delirium; Recent fall with head injury; Chronic decline in memory |
| **Non-delirium group** |
| Recurrent falls, long lie; Vomit; Increasing care needs |
| Fall with long lie; Covid; Cognitive decline |
| Nausea and diarrhoea on background of oesophageal dysmotility and anxiety; Faecal loading; clinical dehydration secondary to diuretics; functional decline at home and anxious about ability to cope at home |
| Fall and head injury; Possible sepsis unknown source - pneumonia vs osteomyelitis; Severe hip osteoarthritis; functional decline; pre-renal Acute Kidney Injury |
| Recurrent falls; rectal cancer; isolated raised C-Reactive Protein; constipation |
| Fall and long lie; Bilateral pleural effusions and pulmonary oedema; Functional decline; Chronic cognitive decline; E.coli UTI |
| Community acquired pneumonia; Atrial fibrillation with rapid ventricular response; Falls |
| Fall and long lie |
| Cognitive decline and low mood; mild Acute Kidney Injury |
| Recurrent falls; Rib fractures |

**Appendix 2: Admission diagnoses**

**Appendix 3: Six-month cognitive and functional outcomes according to delirium status**

|  | **Delirium Arm** | **Non-delirium Arm** | **Overall** |
| --- | --- | --- | --- |
| Died | 2/10 (20%) | 3/10 (30%) | 5/20 (25%) |
| Mild Cognitive Impairment diagnosis | 1 | 1 | 2 |
| Dementia diagnosis | 5 (2 VaD, 1 mixed; 2 subtype not specified) | 6 (5 VaD; 1 mixed) | 11 |
| Concern about cognitive decline | 2 | 0 | 2 |
| New dementia diagnosis | 4/8 | 3/7 | 7/15 |
| New MCI diagnosis | 1/8 | 1/7 | 2/15 |
| Ongoing dementia diagnosis | 1/8 | 2/7 |  |
| Ongoing cognitive concerns not diagnosed | 2/8 | 1/7 | 2/15 |
| Clinical Frailty Scale (mean) | 6.1 | 5.4 | 5.8 |
| Clinical Frailty Scale at 6 months compared with pre-admission | 7 worse  1 unchanged  2 died | 6 unchanged  3 died | 7 worse  7 unchanged  5 died |
| Clinical Frailty Scale at 6 months compared with CFS on scan date | 4 worse  1 improved  3 unchanged  2 died | 4 unchanged  2 improved  3 died | 4 worse  7 unchanged  3 improved  2 died |

**Appendix 4: Six-month cognitive and functional outcomes according to presence or absence of acute infarct on brain MRI**

|  | **New infarct (n=7)** | **No new infarct (n=13)** |
| --- | --- | --- |
| Died | 2/7 (29%) | 3/13 (23%) |
| New dementia or MCI diagnosis | 3/5 (60%) | 6/10 (60%) |
| Ongoing dementia or ongoing cognitive concerns | 2/5 (40%) | 3/10 (30%) |
| Clinical Frailty Scale worse at 6 months compared with pre-admission | 3/5 (60%) | 4/9 (44%) |
